# Alteration of matrix metalloproteinase-3 *O*-glycan structure as a biomarker for disease activity of rheumatoid arthritis

**DOI:** 10.1186/s13075-016-1013-2

**Published:** 2016-05-21

**Authors:** Masaru Takeshita, Atsushi Kuno, Katsuya Suzuki, Atsushi Matsuda, Hiroko Shimazaki, Tomomi Nakagawa, Yuki Otomo, Yasuaki Kabe, Makoto Suematsu, Hisashi Narimatsu, Tsutomu Takeuchi

**Affiliations:** Division of Rheumatology, Department of Internal Medicine, Keio University School of Medicine, 35 Shinanomachi, Shinjuku-ku, Tokyo, 160-8582 Japan; Research Center for Medical Glycoscience (RCMG), National Institute of Advanced Industrial Science and Technology (AIST), 1-1-1 Umezono, Tsukuba, Ibaraki 305-8568 Japan; Center for Systems Medicine, Keio University School of Medicine, 35 Shinanomachi, Shinjuku-ku, Tokyo, 160-8582 Japan; Department of Biochemistry, Keio University School of Medicine, 35 Shinanomachi, Shinjuku-ku, Tokyo, 160-8582 Japan

**Keywords:** Rheumatoid arthritis, Glycoprotein, Biomarker, MMP-3

## Abstract

**Background:**

Nearly all secreted proteins are glycosylated, and serum glycoproteins that exhibit disease-associated glycosylation changes have potential to be biomarkers. In rheumatoid arthritis (RA), C-reactive protein (CRP), and matrix metalloproteinase-3 (MMP-3) are widely used as serologic biomarkers, but they lack sufficient specificity or precision. We performed comparative glycosylation profiling of MMP-3 using a recently developed antibody-overlay lectin microarray technology that allows semicomprehensive and quantitative analysis of specific protein glycosylation to develop an RA-specific disease activity biomarker.

**Methods:**

Serum was taken from patients with RA (*n* = 24) whose disease activity was scored using composite measures, and MMP-3 was immunoprecipitated and subjected to lectin microarray analysis. A disease activity index (DAI) based on lectin signal was developed and validated using another cohort (*n* = 60). Synovial fluid MMP-3 in patients with RA and patients with osteoarthritis (OA) was also analyzed.

**Results:**

Intense signals were observed on a sialic acid-binding lectin (*Agrocybe cylindracea* galectin [ACG]) and *O*-glycan-binding lectins (Jacalin, *Agaricus bisporus* agglutinin [ABA], and *Amaranthus caudatus* agglutinin [ACA]) by applying subnanogram levels of serum MMP-3. ACG, ABA, and ACA revealed differences in MMP-3 quantity, and Jacalin revealed differences in MMP-3 quality. The resultant index, ACG/Jacalin, correlated well with disease activity. Further validation using another cohort confirmed that this index correlated well with several DAIs and their components, and reflected DAI changes following RA treatment, with correlations greater than those for MMP-3 and CRP. Furthermore, MMP-3, which generated a high ACG/Jacalin score, accumulated in synovial fluid of patients with RA but not in that of patients with OA. Sialidase digestion revealed that the difference in quality was derived from *O*-glycan α-2,6-sialylation.

**Conclusions:**

This is the first report of a glycoprotein biomarker using glycan change at a local lesion to assess disease activity in autoimmune diseases. Differences in the degree of serum MMP-3 α-2,6-sialylation may be a useful index for estimating disease activity.

## Background

Rheumatoid arthritis (RA) is a common autoimmune disease characterized by synovial inflammation and hyperplasia. The hyperplastic synovium produces vast amounts of matrix metalloproteinases (MMPs) that degrade cartilage matrix as well as synovial inflammatory cytokines such as macrophage colony-stimulating factor and receptor activator of nuclear factor κB ligand that promote osteoclastogenesis and disrupt joint structure [1].

To prevent RA progression, it is important to define the treatment target, such as remission or at least low disease activity; to assess disease activity using composite measures; and to adapt therapy if the target is not achieved within a particular time frame. This is referred to as a *treat-to-target strategy* [2, 3]. The Disease Activity Score in 28 joints (DAS28), which combines evaluation by a rheumatologist, laboratory test results, and the patient global assessment, has commonly been used to assess disease activity [4, 5]. Recently, new indices such as the Simplified Disease Activity Index [6] and Clinical Disease Activity Index [7], which simplified the DAS28, have been developed.

C-reactive protein (CRP) and MMP-3 are widely measured as serum markers. Although CRP, an acute phase protein, reacts to joint inflammation, it cannot distinguish RA activity and other inflammatory conditions such as infectious disease. In contrast, MMP-3 is characterized as a more specific indicator of synovial inflammation. It was originally identified as a protein secreted from RA synovial fibroblasts [8]. MMP-3 degrades various extracellular substrates, including proteoglycan, fibronectin, laminin, and type 4 collagen, in addition to activating pro-MMPs. Thus, MMP-3 is thought to contribute to cartilage destruction in RA pathophysiology [9]. Serum MMP-3 is elevated in diseases that involve joint synovitis, including RA, reactive arthritis, psoriatic arthritis, and crystal arthritis, but not in osteoarthritis (OA) or systemic inflammatory conditions such as sepsis [10, 11]. However, correlation with disease activity indices (DAIs) is superior in acute phase proteins compared with serum MMP-3 [12, 13]. Thus, development of an RA-specific disease activity biomarker is needed.

It is known that almost all secreted proteins are glycosylated, that glycosylation patterns are influenced by cellular differentiation, and that serum glycoproteins exhibiting disease-associated glycosylation changes have potential to be biomarkers [14]. For example, serum α-fetoprotein (AFP), a commonly used hepatocellular carcinoma biomarker, can be fractionated into three glycosylation patterns—L1, L2, and L3—using *Lens culinaris* agglutinin lectin. Because AFP-L3 is produced only by hepatocellular carcinoma, measurement of AFP-L3 rather than total AFP provides superior sensitivity and specificity [15, 16].

Although analysis of carbohydrate chains has been difficult because of their repetitive sequence and structural variety, the recently developed antibody-overlay lectin microarray technology allows semicomprehensive and quantitative analysis of protein glycosylation patterns [14]. Kuno et al. [17] showed that the glycosylation pattern of serum Mac-2-binding protein, which had previously been reported as a quantitative marker for tumor progression and metastasis [18], gradually changes during liver fibrosis progression and thus serves as a biomarker for liver fibrosis.

In the present study, we focused on an existing biomarker, MMP-3, and examined the association between its glycosylation pattern and RA disease activity. We report on a new, sensitive biomarker that is based on local inflammation and can be assessed using protein glycosylation changes.

## Methods

### Patients and samples

RA serum and synovial fluid samples were collected at Keio University Hospital. All patients fulfilled the 2010 American College of Rheumatology/European League Against Rheumatism classification criteria for RA [19]. Written informed consent was obtained from all individuals. This study was approved by the institutional review board of Keio University School of Medicine and the National Institute of Advanced Industrial Science and Technology, and it was conducted in compliance with the Declaration of Helsinki.

### Sample preparation

Serum MMP-3 enrichment was performed as previously described [14]. Serum or synovial fluid samples were precleared with 100-μg streptavidin beads (FG beads; Tamagawa Seiki, Iida, Japan) at 4 °C for 30 minutes in Tris-buffered saline containing 1 % Triton X-100 (TBS-Tx). Protein G-purified biotinylated anti-MMP-3 antibody (200 ng; R&D Systems, Minneapolis, MN, USA) was added to precleared samples and incubated overnight at 4 °C. Antibody complexes were incubated with 100-μg streptavidin beads at 4 °C for 30 minutes, washed three times with TBS-Tx. MMP-3 and biotinylated anti-MMP-3 antibody were eluted by boiling at 95 °C for 5 minutes in Tris-buffered saline containing 0.2 % sodium dodecyl sulfate. Antibodies were removed by incubation with 100-μg streptavidin beads at 4 °C for 30 minutes. Supernatants were collected and used for the next assay as immunoprecipitation (IP) samples. For sialic acid digestion, IP samples were incubated with Glyko Sialidase A (PROzyme, Hayward, CA, USA) at 37 °C for 1 h before applying them to lectin microarrays (LecChip; GlycoTechnica, Yokohama, Japan).

### Western blot analysis

IP samples and a recombinant MMP-3 protein standard (Abcam, Cambridge, UK) were isolated by 10–20 % SDS-PAGE and transferred onto polyvinylidene difluoride membranes. Membranes were blocked with Block Ace (DS Pharma Biomedical, Osaka, Japan) for 1 h, followed by incubation with biotinylated anti-MMP-3 antibody and streptavidin-HRP conjugate (Jackson ImmunoResearch, West Grove, PA, USA). After extensive washing, MMP-3 bands were semiquantified using ImmunoStar LD (Wako, Osaka, Japan).

### Antibody-overlay lectin microarray

**Fig. 1:**
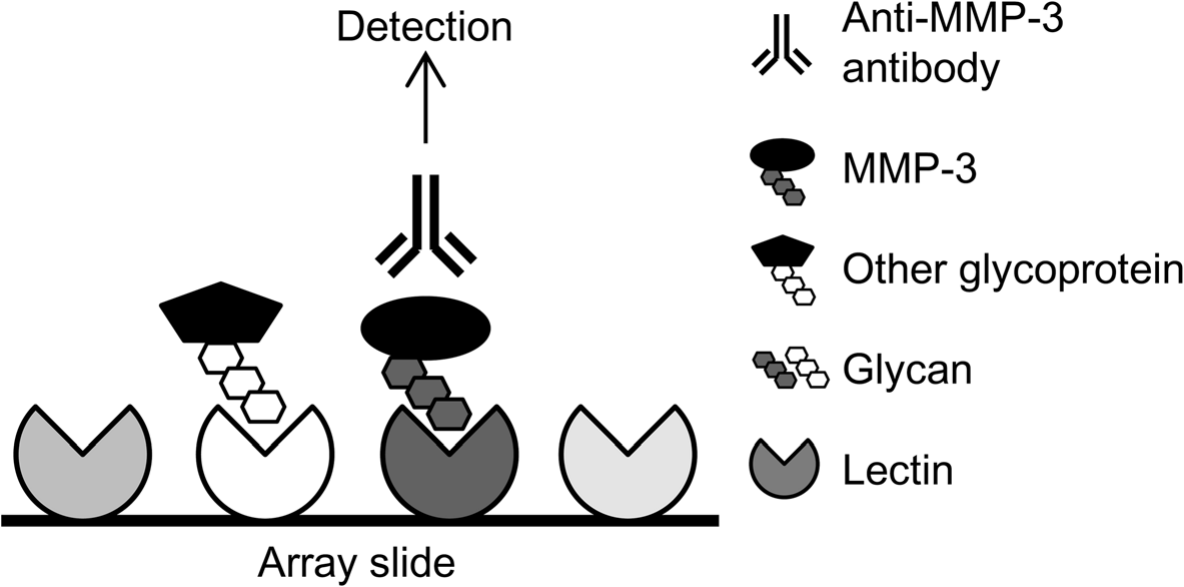
Schematic overview of the antibody-overlay lectin microarray. A total of 45 kinds of lectins are spotted in triplicate on the array slide. Immunoprecipitation samples were applied to them, and glycoproteins bound to the lectins through their glycans. After washing, the lectins that bound to matrix metalloproteinase (MMP)-3 glycans were detected using anti-MMP-3-specific antibody

Antibody-overlay lectin microarray assays were performed as described previously [20, 21]. Briefly, IP samples were diluted to 60 μl with PBS containing 1 % Triton X-100 (PBS-Tx) and applied to lectin microarrays. After incubation overnight at 20 °C, human serum immunoglobulin G (IgG) (2 μl, approximately 10 mg/ml; Sigma-Aldrich, St. Louis, MO, USA) was added to the array, followed by 30 minutes of incubation. The reaction solution was discarded, and the array was washed with PBS-Tx. Biotinylated anti-MMP-3 antibody (100 ng) was applied to the array, incubated at 20 °C for 1 h, washed with PBS-Tx, and incubated at 20 °C for 30 minutes with 200 ng of cyanine 3-labeled streptavidin (GE Healthcare, Little Chalfont, UK). The array was rinsed with PBS-Tx and scanned using an evanescent-field fluorescence scanner (GlycoStation; GlycoTechnica). All data were analyzed with ToolsPro Suite software V1.5 (GlycoTechnica). Net intensity was calculated by subtracting the mean background from the mean signal intensity of three spots per lectin. A schematic overview of this assay is shown in Fig. 1.

### Serum and synovial MMP-3 measurement

Serum MMP-3 was measured using a latex turbidimetric immunoassay (Eiken Chemical, Tokyo, Japan). Synovial fluid MMP-3 was measured by enzyme-linked immunosorbent assay (R&D Systems) according to the manufacturer’s instructions.

### Statistics

Correlation analysis was conducted using the Pearson product-moment correlation coefficient (JMP 11 software; SAS Institute, Cary, NC, USA). We considered *p* < 0.05 to be significant.

## Results

### MMP-3 has an *O*-glycan cluster

**Fig. 2:**
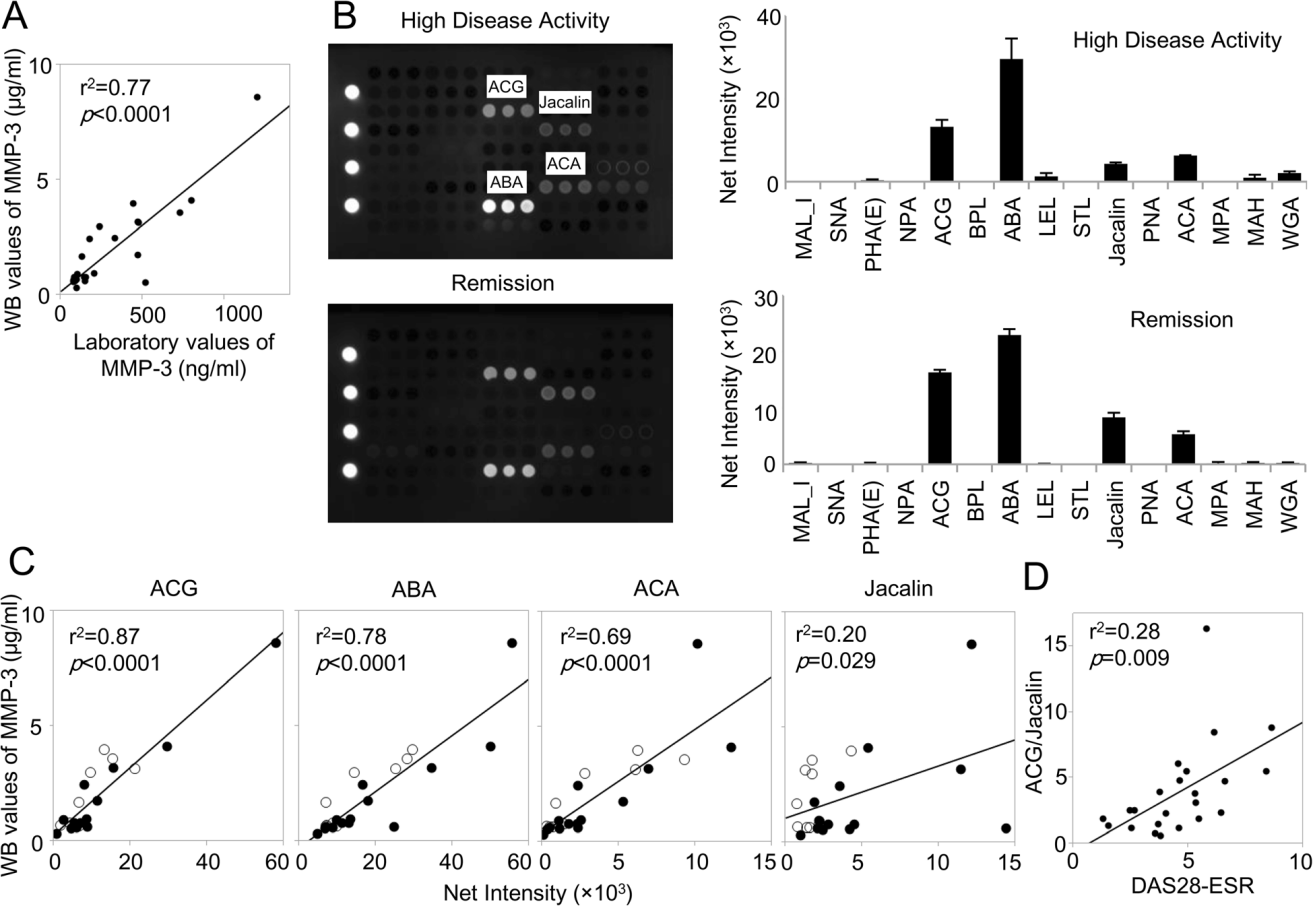
The *Agrocybe cylindracea* galectin (ACG)/Jacalin index reflects rheumatoid arthritis (RA) activity. **a** Correlation of matrix metalloproteinase-3 (MMP-3) quantities before and after immunoprecipitation (IP). Serum MMP-3 measured by turbidimetric immunoassay and MMP-3 in IP samples measured by Western blot (WB) analysis (*n* = 24). **b** Representative data derived from antibody-overlay lectin microarray. Data are from a patient with RA who had high disease activity and a patient with RA who was in remission. Net intensity from triplicate experiments is presented as the mean ± SD. **c** Correlation between immunoprecipitated MMP-3 quantity and lectin signal. Patients with more than moderate disease activity are represented by *open symbols*; otherwise, data are shown as *filled symbols*. **d** Correlation between the ACG/Jacalin index and Disease Activity Score in 28 joints with erythrocyte sedimentation rate (DAS28-ESR). *MAL_I Maackia amurensis* leukoagglutinin I, *SNA Sambucus nigra *agglutinin, *PHA(E) Phaseolus vulgaris *erythroagglutinin,* NPA Narcissus pseudonarcissus* agglutinin, *BPL Bauhinia purpurea* lectin, *ABA Agaricus bisporus* agglutinin, *LEL Lycopersicon esculentum* lectin, *STL Solanum tuberosum* lectin, PNA peanut agglutinin, *ACA Amaranthus caudatus* agglutinin, *MPA Maclura pomifera* agglutinin, *MAH Maackia amurensis* hemagglutinin, WGA wheat germ agglutinin

MMP-3 is a serum protein present in relatively low abundance (10–100 ng/ml in healthy people and 50–1000 ng/ml in patients with RA) [11]. Therefore, MMP-3 was enriched from precleared serum (40 μl) of 24 patients with RA by IP (Table 1). To reduce contaminant, we carefully selected streptavidin beads [22] and used anti-MMP-3 antibody after protein G purification, and removed the antibody from IP eluate. Although small amounts of contaminated protein remained in IP samples, we confirmed that the contaminant did not inhibit lectin signals by spike testing using recombinant MMP-3. The yield and stability of IP samples was checked by Western blot analysis (Fig. 2a), and we confirmed that there were no extra bands in all samples. Because we used the same antibody for Western blot analysis and antibody-overlay lectin microarray experiments, almost all lectin signals were thought to be derived from MMP-3. Aliquots of the IP samples, including approximately 1 ng of MMP-3, were subjected to antibody-overlay lectin microarray.

**Table 1:** Characteristics of the first patient cohort

Characteristic	First cohort (*n* = 24)
Age, years	68 (55–72)
Female, *n* (%)	20 (83)
Disease duration, months	36 (6–153)
Stage I/II/III/IV, *n*	9/8/2/5
Class 1/2/3/4, *n*	5/14/4/0
RF-positive, *n* (%)	19 (79)
ACPA-positive, *n* (%)	20 (87)^a^
Methotrexate use, *n* (%)	13 (54)
Biologic use, *n* (%)	8 (33)
DAS28-ESR	4.6 (3.6–5.8)^a^

*RF* rheumatoid factor, *ACPA* anticitrullinated protein antibodies, *DAS28-ESR* Disease Activity Score in 28 joints with erythrocyte sedimentation rate

Values are median (interquartile range) unless otherwise noted

^a^ *n* = 23

Representative results from two patients, one with high disease activity and the other in remission, are shown in Fig. 2b. Although trace amounts of MMP-3 were applied, four lectins showed significant signal in all samples: *Agrocybe cylindracea* galectin (ACG), *Agaricus bisporus* agglutinin (ABA), *Amaranthus caudatus* agglutinin (ACA), and Jacalin. ACG recognizes α-2,3 sialic acid [23], ABA, ACA, and Jacalin commonly recognize the T antigen (core 1 structure) of *O*-glycan [21]. Such a unique binding pattern at a low concentration has been observed for mucin-like *O*-glycosylated proteins such as mucin 1 [24], podoplanin [21], and immunoglobulin A (IgA) [20]. The estimated glycan structure of the MMP-3 molecule was discussed later in this article. We did not observe a signal for *N*-glycan binding, although MMP-3 has a potential *N*-glycosylation site. It is well known that interaction of a glycoprotein that has a single *N*-glycan, such as AFP, with lectins is weak and difficult to detect at low analyte concentrations. To our knowledge, this is the first report to describe MMP-3 as having an *O*-glycan cluster.

### ACG/Jacalin ratio reflects RA activity

We observed differences between lectin signals among patients with RA, prompting us to next evaluate the association between lectin signal and RA disease activity. Serum MMP-3 levels varied among patients, with the quantity of MMP-3 that was enriched and analyzed, in addition to the strength of individual lectin signals related to serum MMP-3 concentration, differing. Therefore, we evaluated the correlation between representative lectin signals and MMP-3 quantity (Fig. 2c). The results show that ACG, ABA, and ACA signals correlated well with MMP-3, regardless of RA disease activity. This suggests little variation in the structure of ACG, ABA, and ACA binding glycans. Jacalin signal, however, correlated poorly with MMP-3, suggesting that there were variations in the structure of Jacalin-binding glycans. Especially among patients with a high RA disease activity, Jacalin signals seemed to be relatively low compared with quantity of MMP-3; therefore, we thought it might be possible to use Jacalin as the RA activity biomarker.

However, there are two problems with using Jacalin signals as a disease activity marker: (1) lectin signals is influenced by serum MMP-3 concentration; and (2) because the relationship between disease activity and Jacalin signals was negatively correlated, use of Jacalin signals is impractical. To overcome these problems, we took the ratio of the Jacalin and ACG signals, which had the strongest correlation to MMP-3 quantity. As expected, the ACG/Jacalin index was positively correlated with RA disease activity (Fig. 2d). This result suggests that profiling MMP-3 glycosylation patterns produced in local RA lesions may serve as a disease activity marker.

### ACG/Jacalin index as an RA disease activity marker

To confirm the association between the ACG/Jacalin index and disease activity, we recruited a second patient cohort and measured the ACG/Jacalin index in the same manner. This validation cohort included 60 serum samples from 30 patients with RA before and after treatment. The median treatment period was 12 months (interquartile range 11–12 months) (Table 2).

**Table 2:** Characteristics of the second patient cohort

Characteristic	Second patient cohort (*n* = 60)	
	Pretreatment (*n* = 30)	Posttreatment (*n* = 30)
Age, years	56 (37–64)	
Female, *n* (%)	26 (88)	
Disease duration, months	13 (3–47)	
Stage I/II/III/IV, *n*	14/14/0/2	
Class 1/2/3/4, *n*	9/19/2/0	
RF-positive, *n* (%)	22 (73)	
ACPA-positive, *n* (%)	22 (73)	
Methotrexate use, *n* (%)	18 (60)	28 (93)
Biologic use, *n* (%)	5 (17)	21 (70)
DAS28-ESR	4.8 (3.7–6.4)	2.0 (1.0–2.8)
DAS28-CRP	4.0 (3.2–5.5)	1.5 (1.1–2.3)
SDAI	16.0 (11.5–32.9)	1.8 (0.3–5.4)
CDAI	15.4 (10.8–31.2)	1.8 (0.3–5.4)
SJC, 28 joints, *n*	4 (3–8.5)	0 (0–1)
TJC, 28 joints, *n*	6 (3–10)	0 (0–1)
PtGA, mm	48 (27–73)	35 (11–53)
PGA, mm	31 (20–52)	2 (0–11)
CRP, mg/dl	0.5 (0.1–1.2)	0.0 (0.0–0.1)
ESR, mm/h	47 (28–67)	8 (3–24)
MMP-3, ng/ml	79 (49–107)	39 (24–53)
HAQ-DI	0.9 (0.3–1.4)	0.1 (0–0.4)

*ACPA* anticitrullinated protein antibodies, *CDAI* Clinical Disease Activity Index, *CRP* C-reactive protein, *DAS28* Disease Activity Score in 28 joints, *ESR* erythrocyte sedimentation rate, *MMP-3* matrix metalloproteinase-3, *RF* rheumatoid factor, *SDAI* Simplified Disease Activity Index, *SJC* swollen joint count, *TJC* tender joint count, *PtGA* patient global assessment, *PGA* Physician Global Assessment, *HAQ-DI* Health Assessment Questionnaire Disability Index

Values are median (interquartile range) unless otherwise noted

The correlation between composite measures and their components and ACG/Jacalin, MMP-3, and CRP are shown in Table 3. Consistent with previous reports [12, 13], CRP correlated better with DAIs than serum MMP-3. However, focusing on the glycosylation pattern, the ACG/Jacalin index correlated well with disease activity, similar to CRP. This result indicates that the ACG/Jacalin index may serve as a disease activity marker.

**Table 3:** Correlation between disease activity markers, disease activity indices, and their components

	ACG/Jacalin		MMP-3 (females)		CRP	
	*r* ^2^	*p* Value	*r* ^2^	*p* Value	*r* ^2^	*p* Value
DAS28-ESR	0.341	<0.0001	0.196	0.0004	0.309	<0.0001
DAS28-CRP	0.332	<0.0001	0.158	0.0017	0.398	<0.0001
SDAI	0.350	<0.0001	0.131	0.0046	0.399	<0.0001
CDAI	0.353	<0.0001	0.123	0.0059	0.332	<0.0001
SJC, 28 joints, *n*	0.329	<0.0001	0.112	0.0088	0.274	<0.0001
TJC, 28 joints, *n*	0.303	<0.0001	0.104	0.0120	0.333	<0.0001
PtGA, mm	0.153	0.0024	0.064	0.0518	0.119	0.0069
PGA, mm	0.362	<0.0001	0.121	0.0065	0.371	<0.0001
CRP, mg/dl	0.104	0.0138	0.110	0.0098	–	–
ESR, mm/h	0.350	<0.0001	0.283	<0.0001	0.350	<0.0001
MMP-3, ng/ml	0.069	0.0469	–	–	0.110	0.0098
HAQ-DI	0.236	0.0001	0.157	0.0018	0.188	0.0005

*ACG Agrocybe cylindracea* galectin, *CDAI* Clinical Disease Activity Index, *CRP* C-reactive protein, *DAS28* Disease Activity Score in 28 joints, *ESR* erythrocyte sedimentation rate, *HAQ-DI* Health Assessment Questionnaire Disability Index, *MMP-3* matrix metalloproteinase-3, *PGA* Physician Global Assessment, *PtGA* patient global assessment, *SDAI* Simplified Disease Activity Index, *SJC* swollen joint count, *TJC* tender joint count

Pearson product-moment correlation values are shown

**Fig. 3:**
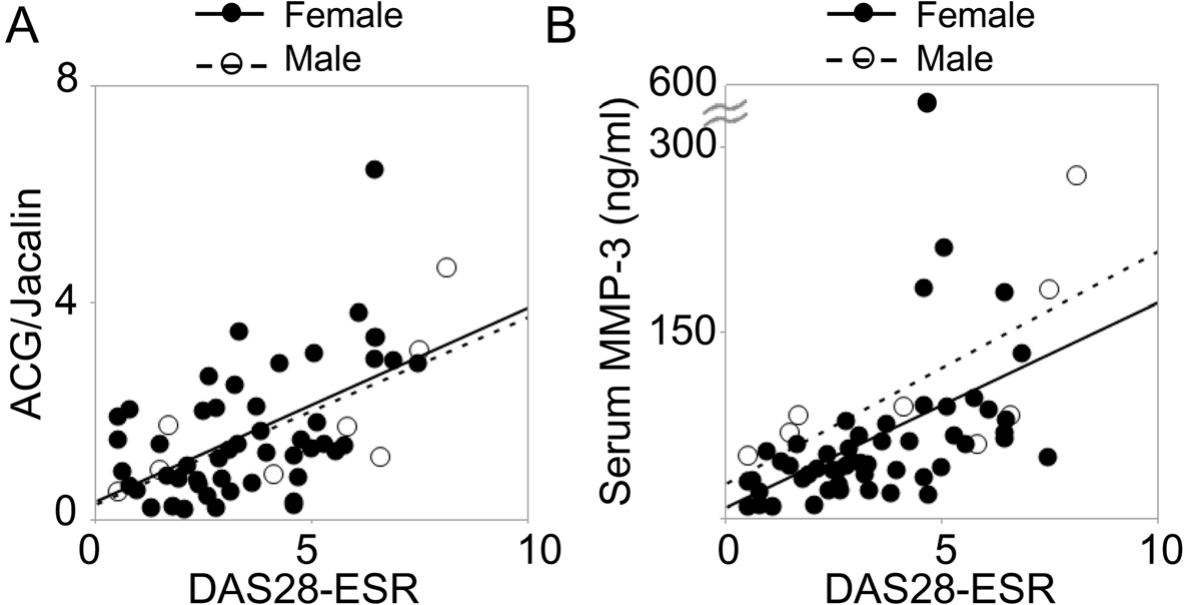
The *Agrocybe cylindracea* galectin (ACG)/Jacalin index is similar between males and females. Correlations between disease activity indices and (**a**) ACG/Jacalin and (**b**) serum matrix metalloproteinase-3 (MMP-3). Data from female and male patients are shown in *filled* and *open symbols*, respectively. *DAS28-ESR* Disease Activity Score in 28 joints with erythrocyte sedimentation rate

Importantly, the ACG/Jacalin index correlated with each component of the composite measures. In particular, the correlation coefficient was high for the joint score and Physician Global Assessment. The correlations with CRP and MMP-3 were not strong, suggesting they may reflect different aspects of disease activity. Because the standard serum MMP-3 value in males and females is different, MMP-3 values were calculated using only female data (Table 3). A gender gap was not observed in the relationship between the ACG/Jacalin index and disease activity (Fig. 3).

To examine whether the ACG/Jacalin index reflects a change in disease activity, we assessed the correlation between the change in the ACG/Jacalin index and DAIs before and after treatment (Table 4). Surprisingly, the ACG/Jacalin index reflected the degree of disease activity change better than MMP-3 and CRP. Similar to previous results, a change in the ACG/Jacalin index was correlated with a change in each component of the composite measures. These results strongly suggest that the ACG/Jacalin index may serve as a useful RA disease activity marker.

**Table 4:** Correlation between the change of disease activity markers, disease activity indices, and their components

	ΔACG/Jacalin		ΔMMP-3 (females)		ΔCRP	
	*r* ^2^	*p* Value	*r* ^2^	*p* Value	*r* ^2^	*p* Value
ΔDAS28-ESR	0.321	0.0017	0.028	0.4114	0.124	0.0564
ΔDAS28-CRP	0.254	0.0062	0.005	0.7334	0.159	0.0292
ΔSDAI	0.245	0.0074	<0.001	0.9379	0.178	0.0203
ΔCDAI	0.241	0.0080	<0.001	0.8952	0.116	0.0651
ΔSJC, 28 joints, *n*	0.174	0.0271	<0.001	0.9215	0.152	0.0330
ΔTJC, 28 joints, *n*	0.180	0.0243	0.003	0.8083	0.109	0.0754
ΔPtGA, mm	0.172	0.0280	0.002	0.8092	0.001	0.8884
ΔPGA, mm	0.215	0.0130	0.006	0.7076	0.170	0.0236
ΔCRP, mg/dl	0.055	0.2293	0.023	0.4553	–	–
ΔESR, mm/h	0.468	<0.0001	0.200	0.0218	0.217	0.0094
ΔMMP-3, ng/ml	0.093	0.1143	–	–	0.010	0.6074
ΔHAQ-DI	0.217	0.0125	0.012	0.5896	0.001	0.9000

*ACG Agrocybe cylindracea* galectin, *CDAI* Clinical Disease Activity Index, *CRP* C-reactive protein, *DAS28* Disease Activity Score in 28 joints, *ESR* erythrocyte sedimentation rate, *HAQ-DI* Health Assessment Questionnaire Disability Index, *MMP-3* matrix metalloproteinase-3, *PGA* Physician Global Assessment, *PtGA* patient global assessment, *SDAI* Simplified Disease Activity Index, *SJC* swollen joint count, *TJC* tender joint count

Pearson product-moment correlation values are shown

### Local RA lesions produce MMP-3 with elevated ACG/Jacalin index

We found that serum MMP-3 glycosylation patterns reflected RA disease activity, and we further examined synovial fluid MMP-3 to confirm that this change was derived from local RA lesions. Synovial MMP-3 was immunoprecipitated from untreated patients with active RA and control patients with OA.

**Fig. 4:**
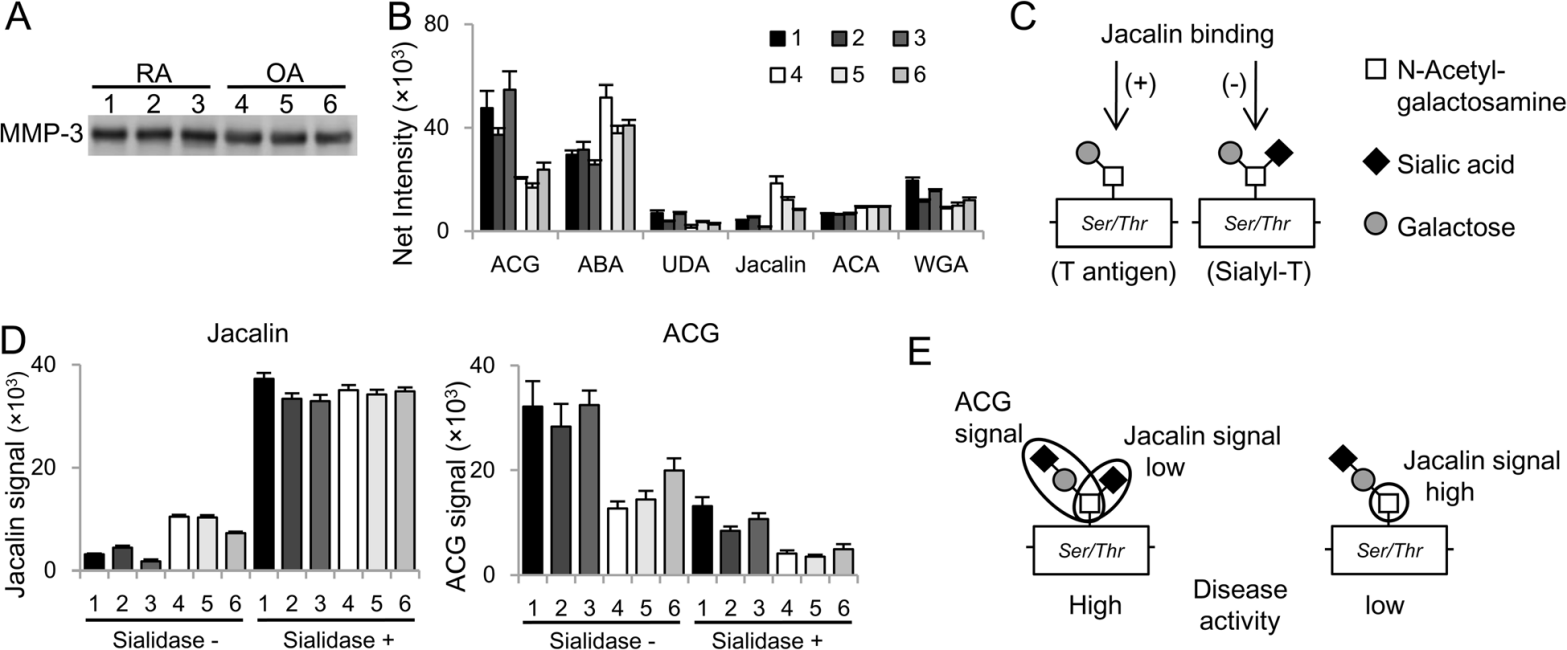
Matrix metalloproteinase-3 (MMP-3) with elevated *Agrocybe cylindracea* galectin (ACG)/Jacalin signals produced in local lesions reflects the degree of sialylation. **a** Synovial fluid MMP-3 was measured by Western blot analysis after immunoprecipitation. Patients with RA (*lanes 1–3*) and patients with OA (*lanes 4–6*). **b** Representative lectin signals derived from synovial MMP-3 (mean ± SD). **c** The structure of T and sialyl-T antigen. Jacalin recognizes the former. **d** Jacalin and ACG signals compared before and after sialic acid digestion (mean ± SD). **e** The estimated basic structure of MMP-3 *O*-glycan. *ABA Agaricus bisporus* agglutinin, *UDA Urtica dioica agglutinin*, *ACA Amaranthus caudatus* agglutinin, *WGA* wheat germ agglutinin

Patient background data and synovial fluid MMP-3 concentrations are shown in Table 5. Synovial MMP-3 was 100-fold higher than serum MMP-3 and higher in RA versus OA, consistent with previous reports [9, 11]. To focus on glycosylation differences, an IP and lectin microarray assay was performed after making MMP-3 concentrations equal by dilution. Synovial IP sample yields were confirmed by Western blot analysis (Fig. 4a), with representative lectin signals from three patients with RA and three patients with OA (Fig. 4b). RA synovial fluid MMP-3 had a high ACG signal and a low Jacalin signal compared with synovial fluid of patients with OA, the same as for high RA disease activity as indicated by serum MMP-3. This result suggests that the increase in the ACG/Jacalin index resulted in part from MMP-3 produced in inflammatory RA lesions.

**Table 5:** Characteristics of patients who provided synovial fluid samples

Patient	Disease	Age (years)	Sex	DAS28-ESR	Synovial fluid MMP-3 (μg/ml)
1	RA	71	Male	5.12	45.5
2	RA	87	Female	6.90	11.0
3	RA	77	Female	4.88	32.0
4	OA	79	Female	–	0.7
5	OA	81	Female	–	1.3
6	OA	67	Female	–	2.4

*DAS28* Disease Activity Score in 28 joints, *ESR* erythrocyte sedimentation rate, *MMP-3* matrix metalloproteinase-3, *OA* osteoarthritis, *RA* rheumatoid arthritis

### ACG/Jacalin reflects the degree of sialylation

Jacalin recognizes the T antigen but does not bind the α-2,6-modification of the core *N*-acetylgalactosamine (GalNAc) structure (Fig. 4c) [21]. Therefore, we hypothesized that the difference in ACG/Jacalin signal might indicate the degree of *O*-glycan α-2,6-sialylation. To confirm this, we analyzed synovial IP samples by antibody-overlay lectin microarray after sialic acid digestion. As expected, this treatment increased Jacalin and decreased ACG signal, with the Jacalin signal being equivalent after digestion (Fig. 4d). ACG recognizes the Sia-α-2,3-Gal-β-1,3 GalNAc structure [23]. Thus, these results indicate that MMP-3 *O*-glycan may have a basic structure (Fig. 4e), with the degree of α-2,6-sialylation on the core GalNAc structure reflected by the ACG/Jacalin ratio.

Taken together, MMP-3 *O*-glycan is highly α-2,6-sialylated in the RA joint lesion, and the ACG/Jacalin index, which reflects changes in sialylation, may serve as a new RA disease activity biomarker.

## Discussion

Abnormal glycosylation has been studied extensively in RA. Studies have shown that RA exhibits an increase in agalactosyl IgG [25]; a decrease in the GalNAc content of IgA [26]; and more recently, extensive glycosylation of the anticitrullinated protein antibodies-IgG variable domain [27]. Notably, most were surrogate markers that were not expressed in diseased synovial tissue. Our data point to a new RA activity marker using a change in glycosylation of a glycoprotein directly expressed in, and secreted from, synovial tissue.

Although CRP and MMP-3 are widely used to monitor RA, they do not demonstrate sufficient specificity to determine disease activity. Thus, RA disease activity markers, such as the multibiomarker disease activity test [28, 29] and the leucine-rich α2 glycoprotein [30], were developed. However, it has been difficult to find a protein that specifically reflects RA disease activity. In this study, we focused on the glycan content of an existing marker, MMP-3, to determine both specificity and sensitivity metrics.

As previously mentioned, glycosylation is influenced by the state of the cell [14]. MMP-3 production is increased in joint synovitis and is continually produced even in noninflamed joints. However, our results show that MMP-3 glycans change under inflammatory conditions and that the ACG/Jacalin index seemed to distinguish disease-specific MMP-3 from total MMP-3. This is one reason why our marker correlated well with disease activity. In addition, MMP-3 expression is relatively restricted within the joint and partly leaks into the serum [11]. Thus, it might be possible to directly determine inflammation at local lesions without an effect of any other influences. Interestingly, the ACG/Jacalin index had a higher correlation coefficient for joint parameters than CRP or MMP-3, suggesting that this index is a more specific arthritis marker.

The ACG/Jacalin index has advantages as a disease activity marker, but further study is required. The method used to obtain lectin signals is slightly complicated, and thus a simplified method is needed. It is better to clarify the glycan structure of MMP-3 and to detect MMP-3 with altered glycan directly. Additionally, sensitivity and stability should be improved for practical use because serum MMP-3 concentration is quite low in some patients after treatment. Although serum MMP-3 was reported to be a radiological predictor [31–33], we could not examine the association between ACG/Jacalin index and radiological progression, owing to the limited number of patients. In addition to RA, MMP-3 glycans may be altered in other disease conditions that increase serum MMP-3, such as psoriatic arthritis, reactive arthritis, steroid use, and polymyalgia rheumatica [10, 31], requiring measurement of ACG/Jacalin under various situations.

To our knowledge, this study shows for the first time that serum glycoproteins exhibiting disease-associated glycosylation changes have potential as disease-specific biomarkers in autoimmune diseases. In patients with RA, differences in serum MMP-3 α-2,6-sialylation may be a useful index for estimating disease activity. Further studies are needed to validate this index and to develop new glycoprotein biomarkers in other autoimmune diseases.

## Conclusions

We have developed a new, sensitive RA-specific biomarker based on MMP-3 glycosylation changes. The ratio of two lectin signals, ACG and Jacalin, correlated well with several DAIs and their components and also reflected DAI changes following RA treatment. Further studies are required to develop the practical use of this biomarker.

## Ethical approval and consent to participate

This study was approved by the institutional review board of Keio University School of Medicine and the National Institute of Advanced Industrial Science and Technology, and it was conducted in compliance with the Declaration of Helsinki. Written informed consent was obtained from the patients for publication of their individual details and accompanying images in this article. The consent forms are held by the authors and are available for review by the Editor-in-Chief of this journal.

## Abbreviations

ABA: *Agaricus bisporus* agglutinin
ACA: *Amaranthus caudatus* agglutinin
ACG: *Agrocybe cylindracea* galectin
ACPA: anticitrullinated protein antibodies
BPL: *Bauhinia purpurea* lectin
AFP: α-fetoprotein
CDAI: Clinical Disease Activity Index
CRP: C-reactive protein
DAI: disease activity index
DAS28: Disease Activity Score in 28 joints
ESR: erythrocyte sedimentation rate
GalNAc: *N*-acetylgalactosamine
HAQ-DI: Health Assessment Questionnaire Disability Index
IgA: immunoglobulin A
IgG: immunoglobulin G
IP: immunoprecipitation
IQR: interquartile range
LEL: *Lycopersicon esculentum* lectin
MAH: *Maackia amurensis* hemagglutinin
MAL_I: *Maackia amurensis* leukoagglutinin I
MMP-3: matrix metalloproteinase-3
MPA: *Maclura pomifera* agglutinin
NPA: *Narcissus pseudonarcissus* agglutinin
OA: osteoarthritis
PBS-Tx: PBS containing 1 % Triton X-100
PGA: Physician Global Assessment
PHA(E): *Phaseolus vulgaris* erythroagglutinin
PNA: peanut agglutinin
PtGA: patient global assessment
RA: rheumatoid arthritis
RF: rheumatoid factor
SDAI: Simplified Disease Activity Index
SJC: swollen joint count
SNA: *Sambucus nigra* agglutinin
STL: *Solanum tuberosum* lectin
TBS-Tx: Tris-buffered saline containing 1 % Triton X-100
TJC: tender joint count
UDA: *Urtica dioica* agglutinin
WGA: wheat germ agglutinin
